# Negative body experience in women with early childhood trauma: associations with trauma severity and dissociation

**DOI:** 10.1080/20008198.2017.1322892

**Published:** 2017-05-31

**Authors:** Mia Scheffers, Maike Hoek, Ruud J. Bosscher, Marijtje A. J. van Duijn, Robert A. Schoevers, Jooske T. van Busschbach

**Affiliations:** ^a^ School of Human Movement and Education, Windesheim University of Applied Sciences, Zwolle, The Netherlands; ^b^ GGZ Centraal, Ermelo, The Netherlands; ^c^ Department of Sociology, University of Groningen, Groningen, The Netherlands; ^d^ Department of Psychiatry, University of Groningen, University Medical Center Groningen, Groningen, The Netherlands; ^e^ Research School of Behavioural and Cognitive Neurosciences (BCN), Interdisciplinary Center for Psychopathology and Emotion regulation (ICPE), Groningen, The Netherlands; ^f^ Rob Giel Research Centre, University of Groningen, Groningen, The Netherlands

**Keywords:** Early childhood trauma, body experience, body attitude, body satisfaction, body awareness, dissociation

## Abstract

**Background**: A crucial but often overlooked impact of early life exposure to trauma is its far-reaching effect on a person’s relationship with their body. Several domains of body experience may be negatively influenced or damaged as a result of early childhood trauma.

**Objective**: The aim of this study was to investigate disturbances in three domains of body experience: body attitude, body satisfaction, and body awareness. Furthermore, associations between domains of body experience and severity of trauma symptoms as well as frequency of dissociation were evaluated.

**Method**: Body attitude was measured with the Dresden Body Image Questionnaire, body satisfaction with the Body Cathexis Scale, and body awareness with the Somatic Awareness Questionnaire in 50 female patients with complex trauma and compared with scores in a non-clinical female sample (*n* = 216). Patients in the clinical sample also filled out the Davidson Trauma Scale and the Dissociation Experience Scale.

**Results**: In all measured domains, body experience was severely affected in patients with early childhood trauma. Compared with scores in the non-clinical group, effect sizes in Cohen’s *d* were 2.7 for body attitude, 1.7 for body satisfaction, and 0.8 for body awareness. Associations between domains of body experience and severity of trauma symptoms were low, as were the associations with frequency of dissociative symptoms.

**Conclusions**: Early childhood trauma in women is associated with impairments in self-reported body experience that warrant careful assessment in the treatment of women with psychiatric disorders.

## 1. Introduction

Early life exposure to trauma, such as prolonged and repeated childhood sexual and physical abuse, has profound and lasting negative effects (Lanius, Vermetten, & Pain, 2010). This type of trauma is typically of an interpersonal nature, includes violation of physical integrity, and occurs at developmentally vulnerable times and under circumstances where escape is not possible (Courtois & Ford, 2009; Herman, 1992). The sequelae of such traumatic experiences are complex and include not only symptoms belonging to the post-traumatic stress disorder (re-experiencing, avoidance/numbing and hyperarousal) but also a range of disturbances in self-regulatory capacities (e.g. Ford, Courtois, Steele, Hart, & Nijenhuis, 2005; Van der Kolk, Roth, Pelcovitz, Sunday, & Spinazzola, 2005).

A crucial but often overlooked impact of early life exposure to trauma is its far-reaching effect on an individual’s relationship with their body (Van der Kolk, 2006, 2014). This subject regained some attention with the neurobiological emphasis on the importance of signalling body-related phenomena in order to enhance self-regulation and control (Van der Kolk, 2006). A central element in narratives of early-traumatized people is their lack of body ownership. The experience of the body as ‘my body’ is acquired in early development and is based on physical experiences and accompanying clear definitions of boundaries between self and others (Straus, 1988). As interpersonal processes between children and the people in their environment are conditions for the construction of a coherent body image, which may be severely impaired by threats to the physical integrity and/or violations of body (Sack, Boroske-Leiner, & Lahmann, 2010). Furthermore, memories of traumatic experiences, which are often body-related, may lead to rejection of and withdrawal from the body, and to a loss of contact with the body (Petzold, 1996). As a consequence, traumatized individuals often find it difficult to attend to inner sensations and perceptions and sometimes even deny any somatic awareness (Price, 2005; Van der Kolk, 2006).

In addition, victims of repeated traumatic events in early life often develop negative body-related attitudes, such as body shame (Andrews, 1997), feelings of disgust or hate towards their body or parts of it associated with trauma (Fallon & Ackard, 2002; Vogt, 2012), body dissatisfaction and lack of body esteem (Wenninger & Heiman, 1998). Furthermore, they frequently report reduced physical vitality and reduced feelings of health (Sack et al., 2010; Wenninger & Heiman, 1998). To summarize, several body-related domains may be negatively affected or damaged as a result of early childhood trauma.

In this study, we use the umbrella term *body experience* (Joraschky, Loew, & Röhricht, 2009; Probst, 1997; Probst, Pieters, Vancampfort, & Vanderlinden, 2008), thereby emphasizing the multidimensionality of the construct. Recent classifications of domains of body experience (Probst et al., 2008; Röhricht et al., 2005) are unanimous in including the following domains: *body attitude*, referring to cognitive, affective and behavioural aspects (Fazio & Olson, 2003); *body satisfaction*, defined as the degree of contentment with appearance or functionality of the body (Alleva, Martijn, Jansen, & Nederkoorn, 2014; Secord & Jourard, 1953), and *body awareness*, defined by Mehling et al. (2009, p. 4) as ‘the perception of bodily states, processes and actions that is presumed to originate from sensory proprioceptive and interoceptive afferents and that an individual has the capacity to be aware of’.

Although problems related to body experience resulting from early childhood trauma seem to be present in more than one domain, the empirical research has predominantly focused on one of the three above mentioned domains at a time. Moreover, comparisons with healthy, non-traumatized individuals are scarce. Only two cross-sectional studies were found using a non-clinical control group; both studies on body satisfaction in sexually abused women (Eubanks, Kenkel, & Gardner, 2006; Wenninger & Heiman, 1998). Eubanks et al. (2006) found body satisfaction, measured with the Mendelson Body-Esteem Scale (Mendelson, Mendelson, & White, 2001), to be significantly lower in the abused group (*n* = 19) than in the non-abused group (*n* = 19). Wenninger and Heiman (1998) reported that women who had been sexually abused in childhood (*n* = 57) scored significantly lower on perceived physical health and on the subscale sexual attractiveness of the Body-Esteem Scale (Franzoi & Shields, 1984) than the control sample (*n* = 47). Dyer et al. (2013) studied differences in body attitude in a group of 84 females in treatment for PTSD related to childhood sexual abuse and in a control group of 53 healthy participants. They used the Dresden Body Image Questionnaire (DBIQ-35; Pöhlmann, Roth, Brähler, & Joraschky, 2014) to measure body attitude, and the Body Image Avoidance Questionnaire (BIAQ; Rosen, Srebnik, Saltzberg, & Wendt, 1991) to assess the frequency with which one engages in avoidance behaviours related to body experience. On both measures, patients reported significantly lower scores than the healthy participants. Borgmann, Kleindienst, Vocks, and Dyer (2014) assessed negative body-related emotions, using the Emotions Rating (ER; Vocks, Legenbauer, Wachter, Wucherer, & Kosfelder, 2007), as well as dissociation, measured by the Dissociation-Distress Scale (DSS-4; Stiglmayr, Schmahl, Bremner, Bohus, & Ebner-Priemer, 2009), in 17 women diagnosed with PTSD after childhood sexual abuse and 24 healthy controls during a standardized mirror confrontation. Patients with PTSD had significantly higher scores on negative body-related emotions as well as on dissociation.

Although confirming the negative effect of early childhood trauma on body experience, the studies show a variety of questionnaires employed to measure body experience with a main focus on either body satisfaction or body attitude. Clinical studies, integrating a wider array of perspectives such as disturbances in body awareness or problems with physical contact and intimacy, are lacking in the area of body experience in trauma. An important question to be answered is whether severity of disturbances in body experience is related to severity of trauma symptoms. If so, the next question is whether their association is as strong as the medium to high correlations found between disturbances in body experience and severity of depression (Gillen, 2015; Kim & Kang, 2015; Röhricht, Beyer, & Priebe, 2002).

Because early childhood trauma is hypothesized to affect body experience in several domains, we consider it worthwhile to obtain more evidence on the negative effects in three specific domains – body awareness, body attitude, and body satisfaction – whose interrelatedness is largely unknown so far.

Another issue hardly addressed in the literature to date is the association between dissociation and domains of body experience. Dissociation is defined by disruption and fragmentation of the usually integrated functions of consciousness, memory, identity, body awareness, and perception of the self and the environment (Lanius, Brand, Vermetten, Frewen, & Spiegel, 2012). It has been suggested that victims of repeated early childhood trauma exhibit more dissociative symptoms than victims of single-incident trauma (Lanius et al., 2012; Vermetten, 2012). In their overview of the effects of early trauma on dissociation, Schauer and Elbert (2010) state that dissociation is directly connected with the body. Price (2005; see also Price & Thompson, 2007) is the sole author addressing dissociation in combination with body awareness in relation to childhood sexual trauma. Therefore, further research into the relationship between dissociation and domains of body experience is needed.

In order to increase our knowledge of body experience in early traumatized people in the manner suggested, we conducted a cross-sectional study in a tertiary care centre for early childhood trauma. The study had three aims: firstly, to explore the level of disturbance in domains of body experience in a group of women with early childhood trauma; secondly, to evaluate the association between different domains of body experience in this same group; and thirdly, to investigate associations between body experience and severity of trauma and levels of psychological dissociation. A better understanding of body-related problems in patients with early childhood trauma may contribute to the development and evaluation of interventions targeting body experience in this group of patients.

## 2. Method

### 2.1. Participants

All 80 patients in treatment at tertiary care centre Transit, GGz Centraal, Ermelo, the Netherlands, a centre specializing in the treatment of clients with a history of early traumatisation, received written as well as oral information about the study. Fifty-three patients, including three men, agreed to participate. For the present study, only women were selected, resulting in 50 women with a mean age of 37.4 (*SD* = 11.8, range 20–60), of whom 21 (42%) received treatment from the outpatient facility, 16 (32%) day treatment and 13 (26%) were inpatients. Of the 27 patients who did not participate, four were advised by their therapist against filling out the questionnaires because of risk of deregulation. Furthermore, 17 patients from the day treatment group did not participate because of logistic issues. Reasons for non-participation of six patients remained unknown.

DSM-IV Axis I diagnoses were established by trained psychologists and confirmed by the resident psychiatrist. Table 1 shows diagnoses and other demographic information of the sample.

**Table 1.** Diagnoses at start of treatment, time in treatment, and demographic information clinical sample (*n* = 50).

		*n*	*%*
Diagnosis^a^	Posttraumatic Stress Disorder	29	58
	Dissociative Identity Disorder	6	12
	Dissociative Disorder Not Otherwise Specified	10	20
	Mood Disorder	4	8
	No diagnosis available	1	2
History of sexual abuse	Yes	40	80
	No	10	20
Time in treatment	< 12 months	26	52
	12–24 months	11	22
	> 24 months	13	26
Level of education	Basic education (≤ 9 years)	9	18
	Secondary education (10–12 years)	24	48
	Higher education (≥ 13 years)	17	34
Work situation	Regular employment	8	16
	Disability pension	28	56
	Other	10	20
	Missing	4	8

^a^according to DSM-IV-TR (American Psychiatric Association [APA], 2000)

Of the 50 women, 40 (80%) reported early childhood sexual abuse during the intake interviews. Five of the 10 remaining patients (20%) reported early childhood sexual abuse in the course of treatment. The other five reported early childhood circumstances such as physical abuse by father, early death of parent, severe alcohol addiction of parent, or growing up in a series of foster homes. Axis II diagnoses were present in 74% of the sample: 16% of the personality disorders were classified as Borderline Personality Disorder, 35% as Personality Disorder Not Otherwise specified and 49% had a deferred diagnosis.

### 2.2. Procedure

The study was approved by the institutional review board of Mental Health Care Central, Ermelo, the Netherlands. All participants signed a written informed consent. Data were collected between November 2012 and March 2013. Participants received an envelope containing a set of general questions regarding demographics, and questionnaires addressing body experience, dissociation and traumatic experiences, to be filled out on-site.

Data from 216 female participants with a mean age of 32.1 (*SD* = 14.5, range 18–65) collected in an earlier study on body experience in a healthy sample (Scheffers et al., 2016) were used to compare the scores on domains of body experience with the group of female trauma patients. Mean age of this sample was significantly lower than that of the clinical sample (*t*(264) = 2.39, *p* = .02).

### 2.3. Measures

**Symptoms of Posttraumatic Stress Disorder (PTSD)** were assessed with the Dutch version of the Davidson Trauma Scale (DTS; Davidson et al., 1997; Sijbrandij, Olff, Opmeer, Carlier, & Gersons, 2008), a self-rating scale that consists of 17 items corresponding with the DSM-IV symptoms for posttraumatic stress disorder (PTSD) (‘Have you been upset by something which reminded you of the event?’; ‘Have you been avoiding doing things or going into situations which remind you about the event?’; ‘Have you had difficulty enjoying things?‘; ’Have you been irritable or had outbursts of anger?’). For each item, the subject rates both frequency (range: 0–4) and severity (range: 0–4) during the previous week. The DTS total score is computed by adding up all frequency and severity items (range: 0–136). Cronbach’s *α* in the present study was .93.

**Dissociative experiences** were measured with the Dissociative Experiences Scale (DES; Bernstein & Putnam, 1986), a 28-item self-report questionnaire assessing the frequency of dissociative experiences. The items relate to experiences of disturbance in identity, memory, awareness and cognition as well as feelings of depersonalization and derealization (‘Some people find that they sometimes are able to ignore pain. Circle a number to show what percentage of the time this happens to you’; ‘Some people find that they sometimes sit staring off into space, thinking of nothing, and are not aware of the passage of time. Circle a number to show what percentage of the time this happens to you’). Subjects circle the percentage of time (given in 10% increments ranging from 0 to 100) they experience the various symptoms. A scale score is obtained by averaging the percentages. Cronbach’s *α* in the present study was .94.

**Body attitude** was assessed using the Dutch version of the Dresden Body Image Questionnaire (DBIQ-35-NL; Scheffers et al., 2016). The DBIQ measures five dimensions of body image: vitality (‘I am physically fit’), body acceptance (‘I wish I had a different body’), self-aggrandizement (‘I use my body to attract attention’), physical contact (‘I do not like people touching me’), and sexual fulfilment (‘I am very satisfied with my sexual experiences‘) (Pöhlmann et al., 2014; Pöhlmann, Thiel, & Joraschky, 2008). Based on the results of Confirmatory Factor Analysis item 33 (‘I like showing my body’), part of the sub-scale acceptance in the original German version was moved to the subscale self-aggrandizement in the Dutch version. Responses are scored on a five-point Likert scale, from ‘not at all’ to ‘entirely’. A higher score means a more positive body attitude. In the present sample, Cronbach’s α was .76 for the subscale vitality, .85 for body acceptance, .78 for self-aggrandizement, .86 for physical contact and .86 for sexual fulfilment.

**Body satisfaction** was measured using the 40-item Body Cathexis Scale (BCS). The BCS was developed by Secord and Jourard (1953), consisting of 53 items and was further developed into a 40-item Dutch scale (Baardman & De Jong, 1984; Balogun, 1986). For each item the client’s satisfaction with some aspect of the body part or function, such as hands, vitality, eyes, coordination, health, and weight, is scored on a five-point scale, ranging from very dissatisfied (1) to very satisfied (5). In a Dutch student sample, validity and reliability were found to be sufficient (Dorhout, Basten, Bosscher, & Scheffers, 2015). For the present sample, Cronbach’s α was .94.

**Body awareness** was assessed with the Somatic Awareness Questionnaire (SAQ; Gijsbers Van Wijk & Kolk, 1996), a Dutch 25-item self-report questionnaire that assesses sensitivity to and awareness of internal bodily signals and states, like energy level (‘I am aware of a cycle in my activity level throughout the day’) and awareness of tiredness (‘I notice distinct body reactions when I am fatigued’). All items were posed as statements addressing awareness of internal bodily processes. Items were scored on a five-point Likert scale (1 = not at all, 2 = barely, 3 = more or less, 4 = somewhat, 5 = very much). In a Dutch student sample, validity and reliability were found to be sufficient (Burger & Gianotten, 2011). In the present sample, Cronbach’s α was .87.

### 2.4. Data analyses

All data analyses were conducted using IBM SPSS Statistics software version 20 and R (R Core Team, 2013). Scale scores were not computed for subjects with at least two missing item scores; DBIQ sub-scales were not computed for subjects with at least one missing item score.

Differences between (sub-)groups were analysed with *t*-tests. Mean differences between subgroups were expressed in Cohen’s *d* and considered large if > .80, moderate between .50 and .79, and small between .20 and .49 (Cohen, 1992). Associations between the variables of interest were analysed by Pearson correlations.

The distributions of the scores were investigated carefully for outliers, which may easily influence both the comparison of means and the computation of the correlation coefficient (see Chen & Popovich, 2002, pp. 55–66). Outliers are likely to occur in the relatively small clinical sample and in view of the expected differences in location of the two samples. Outliers for each (sub-)scale were identified based on boxplots; for pairs of (sub-)scales on scatterplots, and using the Mahalanobis distance for combinations of three and more (sub-)scales. This led to the identification of three outliers in the clinical sample, one with a zero score on the DTS, and the two others with high scores on BCS, SAQ and the sub-scales vitality and body acceptance of the DBIQ. In the non-clinical sample five outliers were identified, three of them in different combinations of low scores on DBIQ (sub-scales vitality and sexual fulfilment), BCS and SAQ, one of them with a high score on SAQ, the last with high scores on DBIQ, BCS and SAQ.

## 3. Results

The mean and standard deviation of the severity of trauma symptoms scale (DTS) were high, revealing a skewed distribution. The mean in the clinical sample was 84.8 (*SD *= 25.8) whereas Davidson, Haresh, Tharwani, and Connor (2002) reported a mean of 64.4 (*SD* = 29.7) for people diagnosed with PTSD. The frequency of dissociative symptoms in this group was 36.6 (*SD* = 17.5). Differences in body experience between the trauma group and the non-clinical group were large and significant (*p* < .001) for all domains (see Table 2). In terms of Cohen’s (1992) criteria effect sizes were largest for DBIQ total mean score and subscale sexual fulfilment (*d* = 2.74 and 2.93 respectively) reflecting the fact that 20 women in the trauma sample obtained the minimum score on sexual fulfilment. When the outliers are omitted from the analysis, the means decrease and increase slightly in the trauma group and non-clinical group, respectively, with overall decreasing standard deviation (see Table 2A in the supplementary material). Thus, the standardized difference between the groups, as measured by Cohen’s *d*, increases for all scale comparisons.

**Table 2.** (Sub)scale score means, standard deviations and standardized differences of the trauma and non-clinical samples.

	Trauma group (*n* = 50)		Non-clinical group (*n* = 216)		
Scale	Mean	SD	Mean	SD	Cohen’s d ^a^
Trauma severity (DTS)	84.8^b^	25.8			
Dissociation (DES)	36.6	17.5			
Body attitude (DBIQ-35)	2.17^d^	0.56	3.58^d^	0.46	2.77
Vitality	2.80	0.78	3.74	0.59	1.36
Body acceptance	2.26	0.96	3.68^c^	0.70	1.69
Sexual fulfilment	1.62^c^	0.71	3.66^d^	0.68	2.93
Self-aggrandizement	1.82	0.61	3.10	0.56	2.18
Physical contact	2.34^c^	0.88	3.80	0.62	1.92
Body Satisfaction (BCS)	2.67	0.59	3.61	0.53	1.68
Body Awareness (SAQ)	2.75 ^c^	0.56	3.14 ^c^	0.47	0.76

DTS = Davidson Trauma Scale; DES = Dissociative Experiences Scale; DBIQ = Dresdner Body Image Questionnaire; BCS = Body Cathexis Scale; SAQ = Somatic Awareness Questionnaire.

^a^ based on unequal variances

^b^ sumscore

^c^ one missing observation

^d^ two missing observations

Table 3 shows Pearson’s correlations between domains of body experience, after removing the outliers in both groups. Correlations vary across (sub-)scales for both the trauma group and the non-clinical group, but are strongest between DBIQ total mean score and BCS, and between subscale body acceptance of the DBIQ and BCS, where the correlations for the trauma group are somewhat higher than for the non-clinical group. Low correlations are found between DBIQ sub-scale vitality and SAQ in both samples and between the four other DBIQ sub-scales with SAQ in the non-clinical sample. Correlations tend to be somewhat lower with influential cases/outliers removed (see Table 3A in the supplementary material for all correlations). Figure 1A in the supplementary material shows the distribution and association of the three body experience scales in both samples where the outliers are indicated.

**Table 3.** Pearson’s correlation coefficients between domains of body experience in the clinical sample and healthy sample after removal of outliers.

	Trauma group (*n* = 47)		Non-clinical group (*n* = 211)	
	BCS (body satisfaction)	SAQ (body awareness)	BCS (body satisfaction)	SAQ (body awareness)
DBIQ (body attitude)	.70^**b^	.46^**c^	.58^**b^	.08^c^
sub-scales DBIQ				
vitality	.37*	.12^a^	.52**	−.12^a^
body acceptance	.77**	.27^a^	.62^*a^	.02^b^
sexual fulfilment	.28^a^	.39^*b^	.34^**b^	.11^c^
self-aggrandizement	.31*	.40^*a^	.33**	.15*^a^
physical contact	.36^*a^	.52^**b^	.17	.15^a^
SAQ	.22^a^		−.02^a^	

BCS = Body Cathexis Scale; DBIQ = Dresden Body Image Questionnaire; SAQ = Somatic Awareness Questionnaire.

* p < .05 ** *p* < .001

^a^ one missing pair of observations

^b^ two missing pairs of observations

^c^ three missing pairs of observations

Associations between trauma severity (DTS), dissociative experiences (DES) and body experience domains were low (see Table 4). Due to the small sample size, the correlations are sensitive to outliers, leading to stronger correlations between DTS and the body experience (sub-)scales (most notably vitality and body awareness), due to the removal of the observations with a zero score on DTS. On the other hand, the correlations of DES with body experience are somewhat lower for the sample with outliers removed, except for those with sexual fulfilment and physical contact. See Table 4A in the supplementary material for correlations before removal of outliers.

**Table 4.** Pearson’s *r* between aspects of body experience (DBIQ, SAQ, BCS), trauma symptoms (DTS) and dissociation (DES) after removal of outliers (*n* = 47).

	DTS	DES
DBIQ (body attitude)	−.31*^c^	−.29^c^
sub-scales DBIQ		
vitality	−.37*^a^	−.06^a^
body acceptance	−.24^a^	−.21^a^
sexual fulfilment	−.17^b^	−.25^b^
self-aggrandizement	−.02^a^	−.20^a^
physical contact	−.13^b^	−.26^b^
SAQ (body awareness)	−.32*^b^	−.31*^b^
BCS (body satisfaction)	−.41*^a^	−.25^a^

BCS = Body Cathexis Scale; DBIQ = Dresden Body Image Questionnaire; DES = Dissociative Experiences Scale; SAQ = Somatic Awareness Questionnaire.

* p < .05

^a^ one missing pair of observations

^b^ two missing pairs of observations

^c^ three missing pairs of observations

## 4. Discussion

The most prominent finding of this study is that body experience is impaired in all domains among women in psychiatric treatment with histories of early childhood trauma compared to women in a community sample. These results confirm findings in earlier studies that trauma that trauma history is associated with both body satisfaction (Wenninger & Heiman, 1998) and body attitude (Dyer et al., 2013; Sack et al., 2010). Sack et al. (2010) reported that women with a background of sexual trauma showed a significantly lower body attitude than non-sexually traumatized patients. The fact that 80% of the women in our sample were sexually traumatized may thus explain the low scores on body attitude. With respect to body awareness, our results provide the first empirical evidence of lower self-reported body awareness in patients with early childhood trauma than in a non-clinical group.

We found an extremely high level of trauma symptoms in the clinical sample. Associations between current level of trauma symptoms and measures of body experience, however, were low. The association between body attitude and body satisfaction was strong, while associations between body attitude and body awareness were medium and those between body satisfaction and body awareness were low. The correlations of the DBIQ sub-scales for body attitude ranged from weak for sexual fulfilment and body satisfaction to strong for body acceptance and body satisfaction. The strong correlation between body acceptance and body satisfaction suggests that overlapping concepts are measured. This is remarkable, since both scales use quite different operationalization: body acceptance is defined broadly, whereas body satisfaction is measured by evaluating specific functions and parts of the body. We conclude that the present study indicates that in patients with an early trauma history domains of body experience, especially body satisfaction and body attitude, are interrelated. The scope of body attitude is, however, wider than that of body satisfaction, because it includes important trauma-related domains such as physical contact, vitality and sexuality. It is therefore advisable to include measures of body attitude when evaluating body experience in trauma. When comparing correlations in the clinical and non-clinical samples, correlations between DBIQ and BCS differ only slightly, whereas correlations between DBIQ and SAQ are higher in the clinical sample than in the non-clinical sample. Interrelatedness of body attitude and body awareness might be higher in the clinical sample because early relational trauma affects the body in a profound and all-encompassing way (Ogden, Minton, & Pain, 2006).

Even though not expressed by high correlations, the frequency of dissociative symptoms was high overall, and coupled with low scores on all domains of body experience. This is in accordance with theoretical models on dissociation (Lanius, 2015; Schauer & Elbert, 2010), where the body, especially body awareness, and dissociation are seen as interrelated. A recent experimental study, using the rubber hand illusion in patients with trauma related dissociation, also provides some evidence for this interrelatedness (Rabellino et al., 2016). The fact that dissociation as operationalized with the DES addresses mainly psychoform dissociation, whereas Nijenhuis (2004) assumes that traumatic experiences do not only lead to psychoform, but also to somatoform dissociative symptoms, such as analgesia, motor disturbances and alternating preferences of tastes and smells, may influence the rather low correlations in our results.

This study has the character of a pilot, it being the first comparison of different domains of body experience between a clinical sample and a non-clinical sample. Even from this perspective, however, the study is limited by the relatively small size of the clinical sample consisting entirely of women. Furthermore, because of the level of non-response and insufficient data on the non-response group, it is not clear to what extent our sample is representative for the patients in treatment at this expert treatment centre. Although trauma history was obtained in intake interviews, our study lacks a formal assessment of trauma history which would have been preferable. The lack of information on somatoform dissociation forms another limitation, especially since sensorimotor dissociative symptoms are thought to be related to serious threat to the integrity of the body (Nijenhuis, 2014; Nijenhuis, van Engen, Kusters, & van der Hart, 2001). Furthermore, the data are cross-sectional which precludes assessing directional associations between symptoms of trauma and dissociation and body experience and the effect of treatment. Due to lack of demographic information on the control group, the comparison between the clinical and non-clinical group was limited. Among other possible biases, women in the control sample may have met criteria for psychiatric disorders or may have been the victims of early childhood trauma.

However, even considering the above limitations, our study gives a clear indication of the far reaching and multidimensional effects of early childhood trauma on a woman’s relationship with her body. Seen from this perspective, it may be advisable to include body experience as a theme in trauma treatment, making use of body-oriented interventions. Similar recommendations have been given in the past, for example by Wenninger and Heiman (1998), who stated the need for development of effective interventions targeting body-related disturbances in the treatment of childhood sexual abuse survivors. Body and movement-oriented interventions are more strongly advocated in recent years, not only from the perspective of self-report measurement of body experience, but also from a neurobiological perspective (Corrigan & Hull, 2015; Ogden & Fisher, 2015; Van der Kolk, 2014). Nevertheless, evidence for body and movement interventions is still scarce (Joraschky & Pöhlmann, 2014; Metcalf et al., 2016) and mainstream interventions for complex trauma are mainly cognitively oriented (Borgmann et al., 2014; Cloitre et al., 2011; Schnyder et al., 2015) and seldom incorporate body-related interventions. An exception is the stabilizing group treatment for complex trauma developed by Dorrepaal et al. (2012), a cognitively oriented programme in which attention is also paid to body experience and sexuality.

Our study not only builds a case for addressing the body in trauma treatment, its results also justify a broader approach than one merely directed at body awareness, including body attitude and body satisfaction as well. Neurobiologists (Kaiser, Gilette, & Spinazzolla, 2010; Van der Kolk, 2014; Warner, Koomar, Lary, & Cook, 2013) state that self-regulation has its basis in a system of psychophysiological reactions and control, and body awareness is essential in this process. Contact with body signals and learning to correctly interpret these signals and react to them are indeed crucial skills. However, enhancing body satisfaction and body attitude may also be considered vital aspects of the treatment process. Disturbed body experience not only refers to lack of contact with the body and its signals, but also to a negative body attitude and a lack of body satisfaction, manifest in total rejection of the body, hating the body and feeling shame about the body, as well as declaring the body or body parts ugly or disgusting. Kremer, Orbach, and Rosenbloom (2013) and Orth (1994) stress the importance of restoring the sense of ownership of the body and regaining control over the body as treatment goals.

Future studies on the disturbance of body experience in trauma should preferably be longitudinal and conducted in larger clinical samples. In order to gain further knowledge on the effects of trauma related events on the level of the body-subject in different trauma populations, it will be helpful to add self-report scales measuring body experience as well as somatic dissociation to the diagnostic instruments already widely used that focus on severity of trauma symptoms and other co-morbid psychopathology.

## Supplementary Material

Supplementary material

## Supplemental material

The supplemental material for this article can be accessed here.
