# Supplementary material for: Negative body experience in women with early childhood trauma: associations with trauma severity and dissociation
Source: Eur J Psychotraumatol. 2017 May 31;8(1):1322892. doi: 10.1080/20008198.2017.1322892 (PMC5475325; doi:10.1080/20008198.2017.1322892)
Supplement: Supplementary material [file zept_a_1322892_sm8318.zip › EJPTScheffers_supplemental mat. Table 3A.docx]

| Table 3A. Pearson’s correlation coefficients between domains of body experience in the clinical sample and healthy sample before removal of outliers. | | | | |
| --- | --- | --- | --- | --- |
|  | Trauma group (*n* = 50) | | Non-clinical group (*n* = 216) | |
|  | BCS (body satisfaction) | SAQ (body awareness) | BCS (body satisfaction) | SAQ (body awareness) |
| DBIQ (body attitude ) | .75^**b^ | .56^**c^ | .53^**b^ | .08^c^ |
|  |  |  |  |  |
| subscales DBIQ |  |  |  |  |
| vitality | .49^*^ | .28^a^ | .48^**^ | -.11^a^ |
| body acceptance | .82^**^ | .44^a^ | .56^*a^ | .05^b^ |
| sexual fulfilment | .27^a^ | .45^*b^ | .33^**b^ | .11^c^ |
| self-aggrandizement | .34^*^ | .35^*a^ | .31^**^ | .15 ^*a^ |
| physical contact | .41^*a^ | .53^**b^ | .20 | .12^a^ |
|  |  |  |  |  |
| SAQ | .36^a^ |  | -.01^a^ |  |

DTS = Davidson Trauma Scale; DES = Dissociative Experiences Scale; DBIQ-35 = Dresdner Body Image Questionnaire; BCS = Body Cathexis Scale; SAQ = Somatic Awareness Questionnaire.

** p* < .05 ** *p* < .001

^a^ one missing pair of observations

^b^ two missing pairs of observations

^c^ three missing pairs of observations
