# Supplementary material for: Negative body experience in women with early childhood trauma: associations with trauma severity and dissociation
Source: Eur J Psychotraumatol. 2017 May 31;8(1):1322892. doi: 10.1080/20008198.2017.1322892 (PMC5475325; doi:10.1080/20008198.2017.1322892)
Supplement: Supplementary material [file zept_a_1322892_sm8318.zip › EJPT Schefferss_Supplemental material Figure 1A.docx]

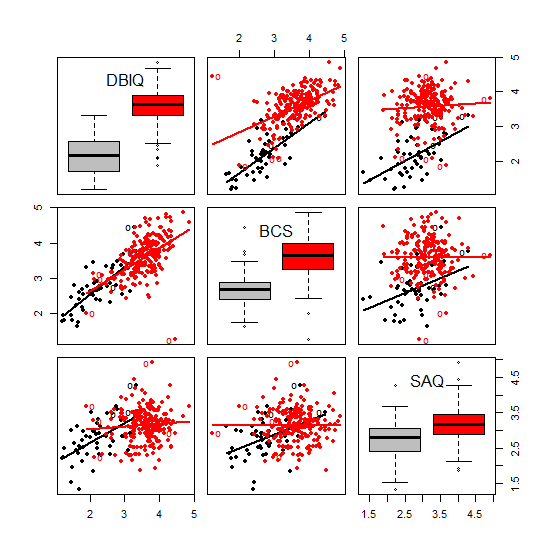


Figure 1A. Boxplots and scatterplots with regression lines for the three body experience scales DBIQ, BCS, and DKB for the trauma sample (*n* = 50, indicated by grey) and the non-clinical sample (*n* = 216, indicated by red), where the outliers (3 in the trauma group, and 5 in the non-clinical sample) are indicated by o.
