# Supplementary material for: Negative body experience in women with early childhood trauma: associations with trauma severity and dissociation
Source: Eur J Psychotraumatol. 2017 May 31;8(1):1322892. doi: 10.1080/20008198.2017.1322892 (PMC5475325; doi:10.1080/20008198.2017.1322892)
Supplement: Supplementary material [file zept_a_1322892_sm8318.zip › EJPTScheffers_supplemental materialTable 4A.docx]

| Table 4A. Pearson’s *r* between aspects of body experience (DBIQ, SAQ, BCS), severity of trauma (DTS) and dissociation (DES) before removal of outliers (*n* = 50). | | |
| --- | --- | --- |
|  | DTS | DES |
| DBIQ (body attitude) | -.18^c^ | -.34*^c^ |
|  |  |  |
| subscales DBIQ |  |  |
| vitality | -.21^a^ | -.17^a^ |
| body acceptance | -.12^a^ | -.28*^a^ |
| sexual fulfilment | -.03^b^ | -.20^b^ |
| self-aggrandizement | -.00^a^ | -.22^a^ |
| physical contact | -.13^b^ | -.26^b^ |
|  |  |  |
| SAQ (body awareness) | -.13*^b^ | -.34*^b^ |
| BCS (body satisfaction) | -.31*^a^ | -.31*^a^ |
| BCS = Body Cathexis Scale; DBIQ = Dresden Body Image Questionnaire; DES = Dissociative Experiences Scale; SAQ = Somatic Awareness Questionnaire.  ** p* < .05  ^a^ one missing pair of observations  ^b^ two missing pairs of observations  ^c^ three missing pairs of observations | | |
