# Supplementary material for: Negative body experience in women with early childhood trauma: associations with trauma severity and dissociation
Source: Eur J Psychotraumatol. 2017 May 31;8(1):1322892. doi: 10.1080/20008198.2017.1322892 (PMC5475325; doi:10.1080/20008198.2017.1322892)
Supplement: Supplementary material [file zept_a_1322892_sm8318.zip › EJPTScheffers et al. suppllem mat. Table 2A.docx]

| Table 2A. (Sub)scale score means, standard deviations and standardized differences of the trauma and non-clinical samples; outliers left out of analysis. | | | | | | | |
| --- | --- | --- | --- | --- | --- | --- | --- |
|  | Trauma group  (*n* = 47) | | | Non-clinical group  (*n* = 211) | | |  |
| Scale | *Mean* | | *SD* | *Mean* | *SD* | | *Cohen’s d* ^a^ |
| Trauma severity (DTS) | 86.3^b c^ | 22.7 | |  | |  |  |
| Dissociation (DES) | 36.4^c^ | 17.2 | |  | |  |  |
|  |  |  | |  | | | |
| Body attitude (DBIQ-35) | 2.13^d^ | 0.52 | | 3.60^d^ | | 0.42 | 3.08 |
| Vitality | 2.76 | 0.72 | | 3.75 | | 0.57 | 1.54 |
| Body acceptance | 2.16 | 0.83 | | 3.69^c^ | | 0.68 | 2.02 |
| Sexual fulfilment | 1.59^c^ | 0.67 | | 3.69^d^ | | 0.61 | 3.26 |
| Self-aggrandizement | 1.82 | 0.62 | | 3.11 | | 0.54 | 2.20 |
| Physical contact | 2.29^c^ | 0.89 | | 3.82 | | 0.60 | 2.01 |
|  |  |  | |  | |  |  |
| Body Satisfaction (BCS) | 2.61 | 0.52 | | 3.64 | | 0.50 | 2.02 |
| Body Awareness (SAQ) | 2.71 ^c^ | 0.52 | | 3.14 ^c^ | | 0.45 | 0.88 |
| DTS = Davidson Trauma Scale; DES = Dissociative Experiences Scale; DBIQ-35 = Dresdner Body Image Questionnaire; BCS = Body Cathexis Scale; SAQ = Somatic Awareness Questionnaire.  ^a^ based on unequal variances  ^b^ sumscore  ^c^ one missing observation  ^d^ two missing observations | | | | | | | |
